# DVF-NET: Bi-Temporal Remote Sensing Image Registration Network Based on Displacement Vector Field Fusion

**DOI:** 10.3390/s25051380

**Published:** 2025-02-24

**Authors:** Mingliang Xue, Yiming Zhang, Shucai Jia, Chong Cao, Lin Feng, Wanquan Liu

**Affiliations:** 1 SEAC Key Laboratory of Big Data Applied Technology, College of Computer Science and Engineering, Dalian Minzu University, Dalian 116600, China; xml@dlnu.edu.cn (M.X.); 202211054029@stu.dlnu.edu.cn (S.J.); 202311054001@stu.dlnu.edu.cn (C.C.); fenglin@dlnu.edu.cn (L.F.); 2 Department of Electronic Engineering, Tsinghua University, Beijing 100084, China; zhang-ym22@mails.tsinghua.edu.cn; 3 School of Intelligent Engineering, Sun Yat-Sen University, Shenzhen Campus, Shenzhen 518107, China

**Keywords:** dual-temporal image registration, displacement vector field, structural attention

## Abstract

Accurate image registration is essential for various remote sensing applications, particularly in multi-temporal image analysis. This paper introduces DVF-NET, a novel deep learning-based framework for dual-temporal remote sensing image registration. DVF-NET integrates two displacement vector fields to address nonlinear distortions caused by significant variations between images, enabling more precise image alignment. A key innovation of this method is the incorporation of a Structural Attention Module (SAT), which enhances the model’s ability to focus on structural features, improving the feature extraction process. Additionally, we propose a novel loss function design that combines multiple similarity metrics, ensuring more comprehensive supervision during training. Experimental results on various remote sensing datasets indicate that the proposed DVF-NET outperforms the existing methods in both accuracy and robustness, particularly when handling images with substantial geometric distortions such as tilted buildings. The results validate the effectiveness of our approach and highlight its potential for various remote sensing tasks, including change detection, land cover classification, and environmental monitoring. DVF-NET provides a promising direction for the advancement of remote sensing image registration techniques, offering both high precision and robustness in complex real-world scenarios.

## 1. Introduction

Remote sensing image registration is a crucial step in spatially aligning multiple remote sensing images acquired at different times, from distinct sensors, or under varying viewpoints. Its primary objective is to align the geometric positions of these images within a unified coordinate system through geometric transformations, thus establishing a foundation for tasks such as image fusion [1,2,3], change detection [4,5,6], 3D reconstruction [7], and other advanced analytical tasks. As a foundational technology in remote sensing, the accuracy and robustness of registration directly influence the performance of downstream tasks. For instance, high-precision change detection depends on accurately aligned multi-temporal images, whereas 3D reconstruction necessitates highly consistent geometric relationships between input images. As a result, research in remote sensing image registration has emerged as a central topic in this field, garnering significant attention from both academia and industry. In response to varying application scenarios and data characteristics, researchers have introduced several registration methods, including feature-based approaches [8,9,10] and methods proposing nonlinear transformations for individual pixels [11,12]. These methods have significantly advanced practical applications in fields such as disaster monitoring, land cover change analysis, urban planning, and environmental protection.

**Figure 1:**
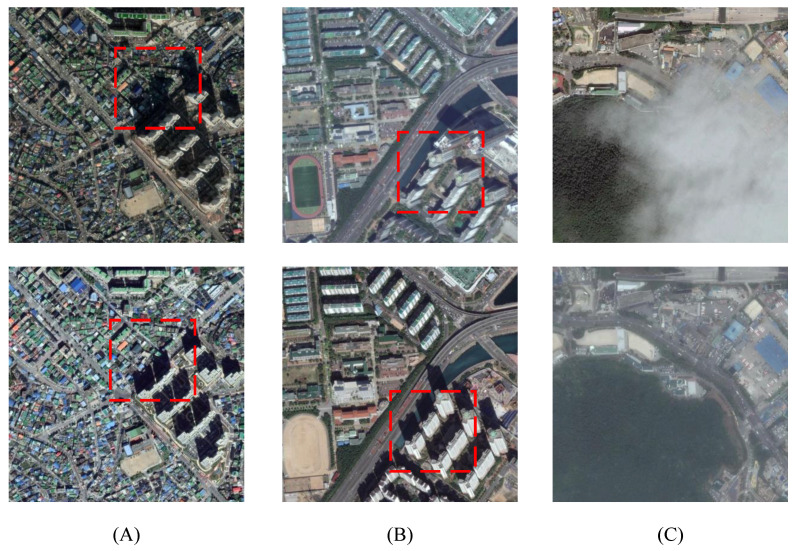
Illustration of some inconsistent factors. The area within the red box shows significant deformation. (**A**) Different image shadow contours caused by different shooting times; (**B**) building tilt caused by different shooting angles; (**C**) obstruction due to rain and fog causes the loss of features.

Despite considerable advancements in remote sensing image registration, several challenges persist. The heterogeneity of multi-source data, the complexity of high-resolution image details, and geometric and radiometric mismatches caused by variations in lighting, viewpoints, and resolution greatly hinder the accuracy and robustness of registration algorithms [13]. On the one hand, due to the effects of land cover changes and the imaging conditions, nonlinear geometric deformation differences often arise between bi-temporal remote sensing images, complicating feature matching. For instance, in the bi-temporal remote sensing images shown in Figure 1, lighting conditions may vary not only in intensity but also in direction [14]. Additionally, changes in the imaging viewpoint often intensify geometric differences [13], and sometimes, interference from rain or fog may cause partial occlusions in certain regions [15]. These complex and dynamic factors intertwine, leading to significant nonlinear geometric deformation differences between the images. Consequently, spatial transformations of different locations in the images during registration exhibit significant inconsistencies, making pixel correspondence extremely complex and challenging to establish. On the other hand, existing remote sensing image registration methods fail to adequately integrate edge structural information of land cover targets. For example, SIFT’s [8] descriptor is based only on local gradient statistics and lacks the incorporation of global structural features such as edge continuity and geometric topology, making it difficult to handle complex nonlinear geometric deformation differences. This limitation results in misalignment in local regions or global geometric distortions in the registration outcomes. Actually, the geometric shape and spatial distribution of land cover targets offer essential clues for describing and constraining nonlinear geometric deformation differences, which is crucial for the model’s understanding of image content. For instance, some semantic segmentation models [16,17,18,19,20] and saliency detection models [21] have significantly enhanced practical performance by integrating edge information to fuse structural features. However, in the field of registration, existing methods tend to focus more on deep feature representation of images and lack effective integration and use of structural information. This neglect of structural information leads to inadequate localization capability for targets, thereby negatively impacting registration accuracy.

**Figure 2:**
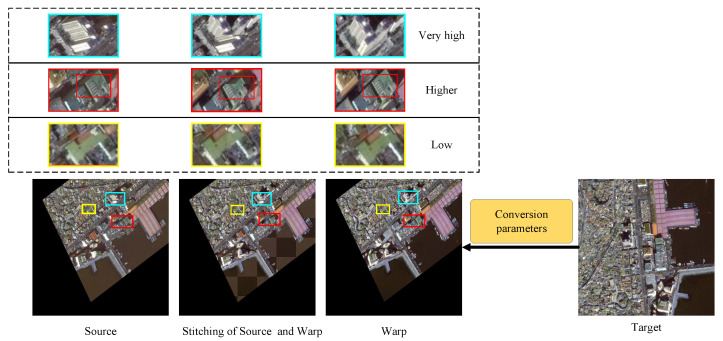
The accuracy of registration varies severely with different building heights. This figure demonstrates the registration results of low, higher, and very high buildings, which are marked by different colors.

Existing methods for addressing nonlinear geometric deformation differences in images are generally divided into two categories: global transformation-based methods and pixel-based methods. First, global transformation-based methods, such as [22,23], utilize precise keypoint pairs to obtain a global transformation matrix, thereby establishing pixel correspondences. However, due to the combined effects of factors such as shooting time intervals, angular differences, and dynamic land cover evolution, the correspondences of local pixels in bi-temporal images no longer align with the main pixels. This inevitably leads to highly complex local nonlinear geometric deformation differences, thereby impacting the accuracy of global transformation-based registration methods. As illustrated in Figure 2, some models perform poorly in registering tall buildings when aligning remote sensing images. These issues influence the accuracy and reliability of registration to some extent. Secondly, pixel-based registration methods, such as [11,12,24], employ structural feature descriptors or pixel displacement in the image as fitting targets to train models, enabling more accurate representation of nonlinear image changes. However, pixel-based registration methods demand a large amount of labeled data for model training. When labeled data are insufficient, these methods often rely on misaligned data as a baseline for image transformation to generate transformation labels for model training. This approach disrupts the original correspondences in the image, thus impacting the accuracy of registration.

The structural information of land cover targets reflects their geometric distribution, which enhances the model’s understanding of remote sensing images and improves the accuracy of instance-level target registration. Structural information in images is commonly employed in image segmentation tasks [16,17] and saliency detection tasks [21]. These methods allow the model to learn and reconstruct edge information from the image, thereby enabling the identification of structural features at a semantic level. However, in image registration tasks, the primary focus is on achieving precise correspondence between the images. In contrast to the emphasis on edge contour information in the aforementioned tasks, the registration task requires the model to accurately identify the object matching relationships between different images. During image registration, relying solely on edge information often fails to fully and accurately capture the complete correspondence of the same object across different images, particularly when dealing with complex backgrounds, occlusions, or deformations, where this issue is further exacerbated.

To address the aforementioned issues, this paper proposes a dual-temporal remote sensing image registration network based on displacement vector field fusion. The proposed network extracts image features through a backbone network incorporating structural feature attention, and it utilizes two transformation matrix estimation modules to fit different geometric transformations in the image, mapping the transformation matrices into displacement vector fields for fusion. First, to address nonlinear geometric deformation differences between images, the network estimates the displacement vector fields by fusing two linear transformation matrices with distinct characteristics, leveraging both affine and projective matrices in registration while also capturing nonlinear geometric deformation differences in the images, thus enabling the network to handle variations in pixel correspondences in bi-temporal images. Second, a structural feature attention module is designed to enable the proposed network to concentrate more on the structural features within the images. By directly utilizing the edge structure map of buildings and fusing the structural correspondences between the two images, the structural attention features generated guide the model to prioritize corresponding structural features during image processing, thereby enhancing the model’s perception and utilization of structural information. Meanwhile, to enable the model to accurately capture correspondences between images, this paper proposes a similarity metric criterion based on the distance between sampled keypoints for supervising model training. By accurately calculating the distance differences between sampled keypoints in different local regions of the images, the model learns the intrinsic correspondence patterns between regions of bi-temporal images, thus better handling the impact of nonlinear factors in the registration task.

**Contributions:** The primary contributions of this paper are as follows:

(1) A remote sensing image registration network is proposed, which combines affine transformation and projective transformation mechanisms to fit different spatial transformations in the image, thereby improving the accuracy of the proposed network when handling nonlinear deformations.
(2) A structural attention module is designed to emphasize the corresponding structural edge information between images, thereby enhancing the model’s ability to extract image features and recognize corresponding relationships between image structures.
(3) A novel method for generating image pairs is proposed, accompanied by a new loss function that utilizes the distance between identical feature points in the two images as a similarity measure. This approach more accurately captures the geometric relationships between corresponding feature points, thereby improving the precision of the correspondence between the images.

## 2. Proposed Approach

### 2.1. Problem Description

Suppose there is a pair of images $I_{S}\in R^{H\times W\times 3}$ and $I_{T}\in R^{H\times W\times 3}$, that need to be aligned. One of these is the target image $I_{T}$, which has correct geographical coordinates for each pixel, while the other is the source image, $I_{S}$, which may exhibit geometric distortions due to factors such as shooting angle, lens distortion, or scene motion.The goal of correcting $I_{S}$ is to determine the optimal transformation parameters *M* that can align the pixel coordinates of the source image $I_{S}$ with those of the target image $I_{T}$. These parameters may include rotation angle, scaling ratio, translation offset, and possibly nonlinear transformation components. This process ensures that both images are consistent in either geographical or geometric terms, resulting in precise image registration and alignment. The alignment process can be expressed mathematically as follows:

(1) $$\begin{array}{c} \mathrm{Min}\left(I_{T}-F_{\mathrm{warp}\;}\left(I_{S},M\right)\right), \end{array}$$

where Min(.) represents minimizing the difference between the target image and the transformed source image, while $F_{\mathrm{warp}\;}\left(.\right)$ represents the operation of transforming the image according to the corresponding transformation parameters.

### 2.2. Overview

**Figure 3:**
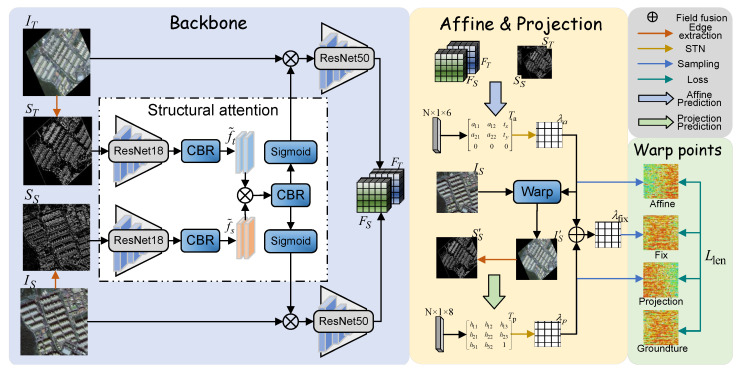
The overall framework of the proposed network, consisting primarily of three parts: the backbone and two displacement vector field prediction modules. These output an affine matrix with 6 degrees of freedom and a projection matrix with 8 degrees of freedom, respectively. $\lambda_{a}\in R^{H\times W\times 2}$ and $\lambda_{p}\in R^{H\times W\times 2}$ correspond to displacement vector fields for these matrices. STN (Spatial Transformer Network) [25] represents the specific spatial transformation network.

As show in Figure 3, the proposed image registration network, DVF-NET consists of three key components. First, there is a backbone module with structural attention, which is used to extract image features. Specifically, the edge extraction algorithm is applied to accurately extract features containing edge structures in the image, and the structural edge images $S_{T}\in R^{H\times W\times 1}$ and $S_{S}\in R^{H\times W\times 1}$ are generated. Subsequently, they are fed into ResNet18 to extract features and perform a dot product operation with the original image according to the following formula. Then, the image features are further extracted by ResNet50, and $F_{S}\in R^{H/4\times W/4\times 512}$ and $F_{T}\in R^{H/4\times W/4\times 512}$ are produced. Secondly, there are two displacement field output networks: the affine transformation displacement field $\lambda_{a}\in R^{H\times W\times 2}$ and the projective transformation displacement field $\lambda_{p}\in R^{H\times W\times 2}$. They receive the features output by the backbone and generate displacement vector fields. Finally, the two displacement vector fields obtained in the previous step are fused to obtain the final displacement vector fields, $\lambda \in R^{H\times W\times 2}$.

(2) $$\begin{array}{c} F_{\alpha }=X_{\alpha }⊗\mathrm{Sigmod}\left({\tilde{f}}_{\alpha }\right) \end{array}$$

Here, $F_{\alpha },\alpha \in \left\{S,T\right\}$ represents the output features, $I_{\alpha },\alpha \in \left\{S,T\right\}$ represents the input source images and target images, ${\tilde{f}}_{\alpha }$ denotes the features extracted by ResNet18, and ⊗ denotes matrix multiplication.

### 2.3. Transform Parameter Prediction

This paper combines two linear transformation matrices to model nonlinear deformations in dual-temporal remote sensing images using two key steps: affine matrix prediction and projection matrix estimation.

**Figure 4:**
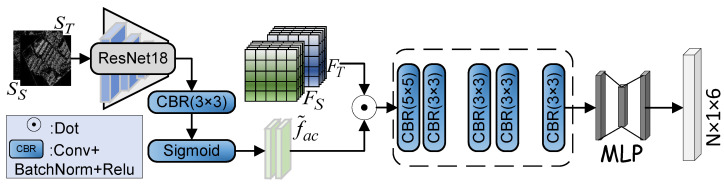
The affine matrix prediction phase. Given the source image feature $F_{S}$, and the target image feature $F_{T}$, along with their respective structural image features, the process outputs an affine matrix $T_{a}$ with 6 degrees of freedom.

*Affine matrix prediction:* Figure 4 illustrates affine matrix prediction. To better utilize the structural information features of the image, this further integrates the edge features into the network, enhancing the module’s structure-aware capabilities and thus yielding more accurate registration results. The features are extracted from the structural edge images $S_{S}$ and $S_{T}$ using ResNet18, and their dimensions are then adjusted to match the features $F_{S}$ and $F_{T}$ extracted by the backbone. Subsequently, sigmoid is used to generate probability maps that guide the model’s attention to structural features. Finally, we generate the transformation parameters $T_{a}$ with 6 degrees of freedom, which can be obtained as follows:

(3) $$\begin{array}{c} T_{a}=\Phi_{a}\left(\mathrm{Cat}\left(F_{S},F_{T}\right)⊙{\tilde{f}}_{ac}\right), \end{array}$$

where $\Phi_{a}\left(.\right)$ represents convolution and fully connected operations, $F_{S}$ and $F_{T}$ denote features extracted from the source and target images, respectively, ⊙ signifies the dot product by element, ${\tilde{f}}_{ac}$ represents the edge structure features in the affine prediction process, and Cat(.) represents a concatenation operation.

**Figure 5:**
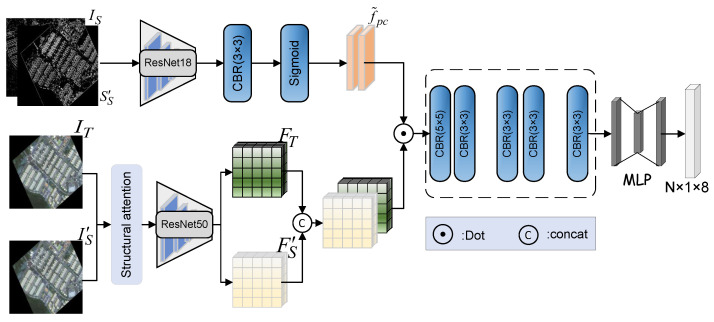
The projection matrix prediction phase. Given the source image feature $F_{S}$ and the target image feature $F_{T}$, along with their respective structural image features, the process outputs a projection matrix $T_{a}$ with 8 degrees of freedom.

*Projection matrix estimation:* As illustrated in Figure 5, to better utilize the affine prediction results, STN is employed to map the transformation parameters $T_{a}$, obtained in the first part, to a displacement vector field $\lambda_{a}\in R^{H\times W\times 2}$. This field $\lambda_{a}$ is then applied to the source image as part of the projection matrix prediction module to obtain the converted source image $F_{S}^{\prime }$. The process of this module is similar to that of the affine prediction module, except that it includes an additional step of image transformation, feature extraction, and edge image extraction. The final output is the projection matrix $T_{p}$, which uses STN network mapping as $\lambda_{p}$. The computational formula is as follows:

(4) $$\begin{array}{c} F_{S}^{\prime }=f_{\mathrm{backbone}}\left(\mathrm{warp}\left(I_{S},\lambda_{a}\right)\right), \end{array}$$

(5) $$\begin{array}{c} T_{p}=\Phi_{g}\left(\mathrm{Cat}\left(F_{S}^{\prime },F_{T}\right)⊙{\tilde{f}}_{pc}\right), \end{array}$$

where $\Phi_{g}\left(.\right)$ represents convolution and fully connected operations, warp(.) refers to the image warping operation, $\lambda_{a}$ denotes the affine matrix, $f_{\mathrm{backbone}}$ is the image features extracted by the backbone network, $F_{S}^{\prime }$ represents the features of the transformed image, ${\tilde{f}}_{pc}$ represents the edge structure features in the projection prediction process, and Cat(.) represents a concatenation operation.

### 2.4. Transform Field Fusion Module

In the final displacement vector field fusion module of the network, we combine the two obtained affine displacement vector fields $\lambda_{a}$ and the projection displacement vector field $\lambda_{p}$ to obtain the fused displacement field $\lambda_{fix}\in R^{H\times W\times 2}$. This field is then applied to the source image. Specifically, we derive two transformation parameters: the affine matrix and the projection matrix. To merge these parameters into a displacement vector field format, we employ the STN. During the fusion process, we subtract the projection displacement vector field $\lambda_{p}$ from the base grid $\lambda_{B}\in R^{H\times W\times 2}$ to obtain the displacement for each individual pixel. This displacement is then added to the affine displacement vector field $\lambda_{a}$ to yield the final displacement vector field $\lambda_{fix}$. Finally, we perform image sampling and interpolation based on the displacement vector field to finally convert the image $I_{C}$. The computational formula is as follows:

(6) $$\begin{array}{c} I_{C}=\mathrm{SaI}\left(\lambda_{a}+\left(\lambda_{p}-\lambda_{B}\right),I_{S}\right), \end{array}$$

where $I_{C}$ represents the transformed source image, SaI(.) denotes the sampling and interpolation operations, $I_{S}$ stands for the source image, and $\lambda_{B}$ denotes the base grid.

### 2.5. Loss Function

DVF-NET integrates transformation parameters within a unified framework, and its training process consists of two main parts: prediction of transformation parameters and final fusion of displacement vector fields. In order for affine prediction and projection prediction to be able to effectively handle different geometric transformations in the image, this paper designs separate loss functions to guide network training:

1. For affine and projection matrices, L1 loss is used as the loss function paradigm. This is because, in manually annotated datasets, errors may exist, and L1 loss is more robust to outliers. L2 loss penalizes larger errors more heavily, which can cause outliers to have an excessive impact on the overall loss, thereby affecting registration accuracy. This loss measures the difference between keypoints in the source and target images within the displacement vector field output by the network. We apply this loss function during the generation of each displacement vector field. The computational formula is as follows:
  (7) $$\begin{array}{c} L_{\mathrm{len}\;}=\frac{1}{3n}\sum_{j=1}^{3}\sum_{i=1}^{n}\left|\varphi_{j}\left({x_{s}}^{i},{y_{s}}^{i}\right)-\left({x_{t}}^{i},{y^{i}}_{t}\right)\right| \end{array}$$
  where $\varphi_{1}\left(.\right)$, $\varphi_{2}\left(.\right)$, and $\varphi_{3}\left(.\right)$ denote the mapping relationships for the affine displacement vector field, the projection displacement vector field, and the fused displacement vector field, respectively. In addition, $\left({x_{s}}^{i},{y_{s}}^{i}\right)$ and $\left({x_{t}}^{i},{y^{i}}_{t}\right)$ represent the corresponding keypoint coordinates of the source and target images.
2. To achieve optimal similarity, DVF-NET introduces a bidirectional loss $L_{ts}$. Specifically, we consider transformations in both directions simultaneously to mitigate biases introduced by unidirectional registration. During the generation of affine and projection displacement vector fields, our network obtains forward transformation matrices λ and inverse transformation matrices $\lambda^{-1}$ and generates forward displacement vector fields Γ and backward displacement vector fields $\Gamma^{-1}$. In an ideal scenario with perfect network estimation, the product of these matrices will closely resemble the identity matrix. This implies that bidirectional transformations can neutralize each other, thereby ensuring registration consistency. Based on this formulation, we define the $L_{ts}$ loss to measure bidirectional registration. The calculation of $L_{ts}$ is as follows:
  (8) $$\begin{array}{c} L_{ts}=\frac{1}{2}\sum_{j=1}^{2}\left|\lambda_{j}⊗\lambda_{j}^{-1}-E\right|, \end{array}$$
  where *E* denotes the identity matrix.
3. At the same time, to balance the errors generated during data labeling, Normalized Mutual Information (NMI) [24] is added to the loss function. NMI leverages the information divergence between the two images to guide the network, thereby improving its accuracy. The loss function $L_{nmi}$ is defined as follows:
  (9) $$\begin{array}{c} L_{\mathrm{nmi}}=\frac{\sum_{a}P_{A}\left(a\right)\log P_{A}\left(a\right)+\sum_{b}P_{B}\left(b\right)\log P_{B}\left(b\right)}{\sum_{a}\sum_{b}P_{AB}\left(a,b\right)\log P_{AB}\left(a,b\right)}, \end{array}$$
  where A and B are a pair of images, $P_{A}\left(a\right)$ and $P_{B}\left(b\right)$ represent the edge probability distributions of individual images, and $P_{AB}\left(a,b\right)$ denotes the joint probability distribution of the two images. When images A and B are aligned, their similarity will be maximized. Therefore, the total loss of the network is as follows:
  (10) $$\begin{array}{c} L_{\mathrm{all}\;}=\alpha_{1}L_{\mathrm{len}\;}+\alpha_{2}L_{ts}+\alpha_{3}L_{nmi}, \end{array}$$
  where $\alpha_{1}$, $\alpha_{2}$, and $\alpha_{3}$ are the weight coefficients.

## 3. Experiment and Data Processing

### 3.1. Datasets

This paper evaluates our method on multiple datasets, including the Google Earth dataset [26] proposed by Park et al., which contains large-viewpoint-offset dual-temporal remote sensing images, and the S2looking dataset [27] with viewpoint deviations. DVF-NET is evaluated separately on both datasets.

1. Google Earth dataset: This dataset consists of 9000 images captured in 2015, 2017, and 2019 by sensors such as Landsat-7, Landsat-8, WorldView, and QuickBird. The images were taken from fixed viewpoints without any restrictions on the imaging angle. For this study, we selected 2500 images from the dataset, using 2000 for training and 500 for testing. This dataset exhibits significant variation, including differences in imaging angles and heights, resulting in considerable nonlinear geometric deformation between the images.
2. S2looking: This dataset includes 5000 images capturing multi-temporal remote sensing data within a three-year period. The dataset imposes restrictions on imaging angles, with an average absolute value of 9.86° and a standard deviation of 12.197°. Similarly, we choose 2500 images from this dataset, with 2000 for training and 500 for testing. The imaging conditions in these data are under control, and the degree of nonlinear geometric distortion is relatively smaller than that of the Google Earth Dataset.

### 3.2. Generation of Image Pairs

**Figure 6:**
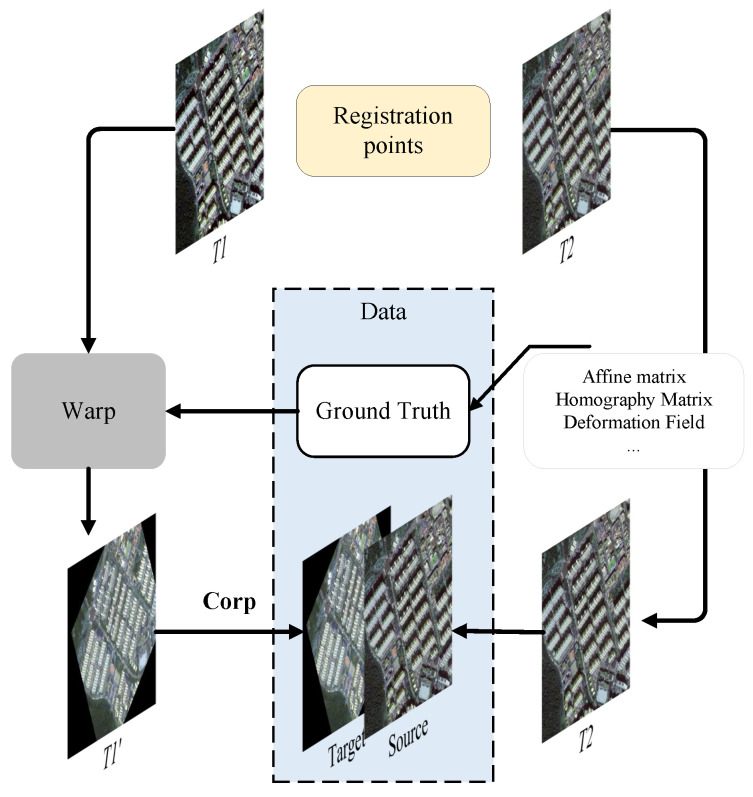
An overview of image pair generation is shown, with the blue section representing dataset construction and the yellow section indicating keypoint extraction.

Comprehensive and sufficient training data are prerequisites for achieving optimal training results. However, in the context of remote sensing image registration, it is challenging to find perfectly corresponding image pairs due to significant inconsistencies between them (such as differences in shooting angles, seasonal variations, and weather conditions). These inconsistencies inevitably lead to errors in the resulting training outcomes. To mitigate this issue, this study improves the image pair generation process by incorporating keypoint coordinates that reflect real-world object positions. The data construction process involves two time-phase images, denoted as $T_{1}$ and $T_{2}$. The details are shown in Figure 6. By applying random affine transformations to $T_{1}$, including rotation, translation, scaling, and shearing, we generate a transformed image $T_{1}^{\prime }$. The scaling parameter is confined to the interval [0.5, 2], the translation values are bounded within [−0.1, 0.1], the rotation angles are limited to [−π, π], and the shearing angles are restricted to the range of [−π/6, π/6]. This approach generates a large amount of data [28]. Additionally, we incorporate keypoint coordinates from the two time-phase images, resulting in a set of *n* coordinate pairs.

### 3.3. Evaluation Metrics

To reveal the effectiveness of the proposed method, we compare it with existing methods, i.e., SIFT [8], LoFTR [29], Roma [23], and DAM-NET [26], on two datasets. SIFT represents a traditional algorithm, while LoFTR and Roma are feature-based image registration methods, and DAM-NET is a region-based remote sensing registration method. The Probability of Correct Keypoints (PCK) and Root Mean Square Error (RMSE) are taken as metrics to evaluate the building alignment quality. The specific calculation formulas are as follows:

(11) $$\begin{array}{c} PCK=\frac{\mathrm{Count}\left(\sum_{i=1}^{n}\left[\sqrt{\alpha_{i}^{2}-P_{i}^{2}}<\tau \right]\right)}{n}, \end{array}$$

(12) $$\begin{array}{c} RMSE=\frac{1}{n}\sum_{i=1}^{n}\sqrt{\alpha_{i}^{2}-P_{i}^{2}}, \end{array}$$

where $P_{i}$ represents the ground truth coordinates, $\alpha_{i}$ denotes the predicted coordinates, and *n* is the number of keypoints. The threshold τ is set to 10 or 8 (as specified in this study), and Count(·) calculates the number of points that satisfy the given condition.

### 3.4. Implementation Details

For the experimental setup, we implemented the proposed network using PyTorch v.1.8.1, which is created by Facebook’s AI Research lab based in Menlo Park, California, USA. The model was trained on an RTX A4000 GPU with 16 GB of VRAM for 100 epochs using the Adam optimizer [30], taking 30 h to complete. The learning rate was set to 0.0001, and the batch size was 4. The dual-temporal remote sensing images used for training had a size of 1024 × 1024 pixels. During the training of DVF-NET, we employed a stepwise approach. Initially, we trained the component responsible for predicting affine displacement vector fields. Once this part was trained, we froze its parameters and proceeded to train the component that predicts perspective displacement vector fields. In the final step, we jointly trained both components, ensuring consistent loss function paradigms throughout.

## 4. Experimental Results

### 4.1. Data Analysis

Table 1 presents the results of the quantitative analysis, where we computed one-way PCK and RMSE from the source image to the target registered image. SIFT is a traditional feature-based registration method. As shown in Table 1, traditional methods have limitations in handling multi-temporal remote sensing images, especially those acquired at two different time points with significant geometric and radiometric differences. LoFTR and Roma are deep learning-based feature registration methods, which demonstrate superior performance compared to SIFT in the registration of multi-temporal images, as shown in Table 1. DAM is a region-based registration method that uses conventional data processing techniques but performs poorly on images with tilted buildings. By comparing the experimental results of the two datasets, we found significant differences in how different registration methods respond to the presence of tilted buildings in images. Additionally, among different deep learning-based methods in the S2looking dataset, the results show minimal variation. Table 1 clearly indicates that our proposed DVF-NET achieved the best registration performance for images with tilted buildings.

#### 4.1.1. Visual Analytics of Results

**Figure 7:**
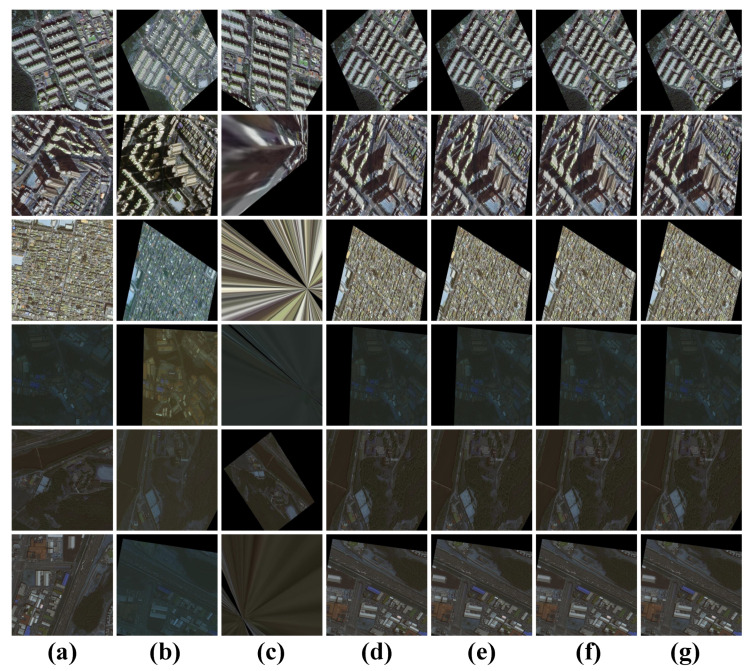
Registration visualization results: (**a**) source; (**b**) target; (**c**) SIFT; (**d**) Lofter; (**e**) Roma; (**f**) DAM-NET; (**g**) DVF-NET. SIFT encountered global misalignment, while the other methods could all complete the registration.

**Figure 8:**
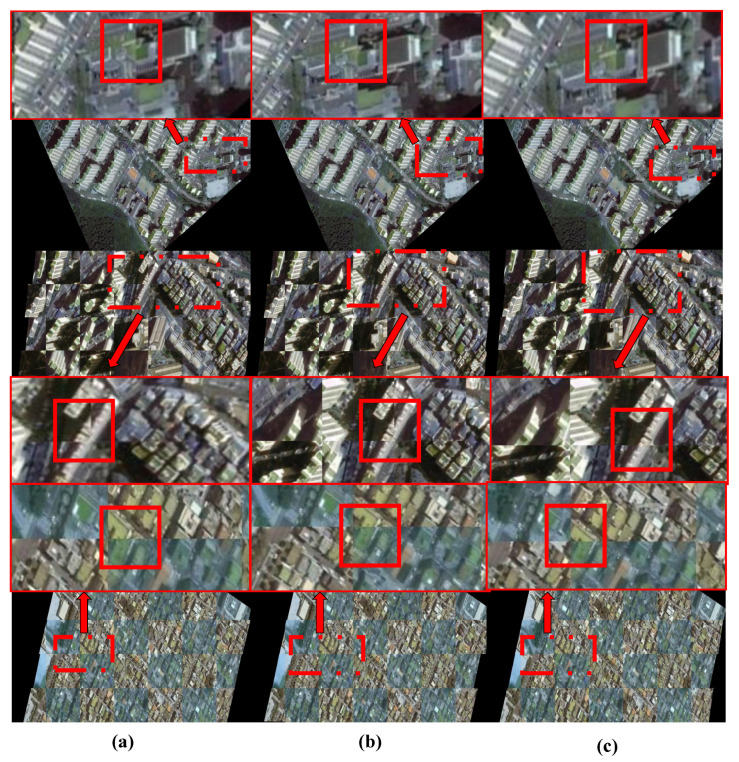
Local comparison of feature-based and region-based models with DVF-NET on Google Earth dataset. The solid red box shows the stitching effect of the converted image and the target image: (**a**) Roma; (**b**) DAM-NET; (**c**) DNF-NET.

**Figure 9:**
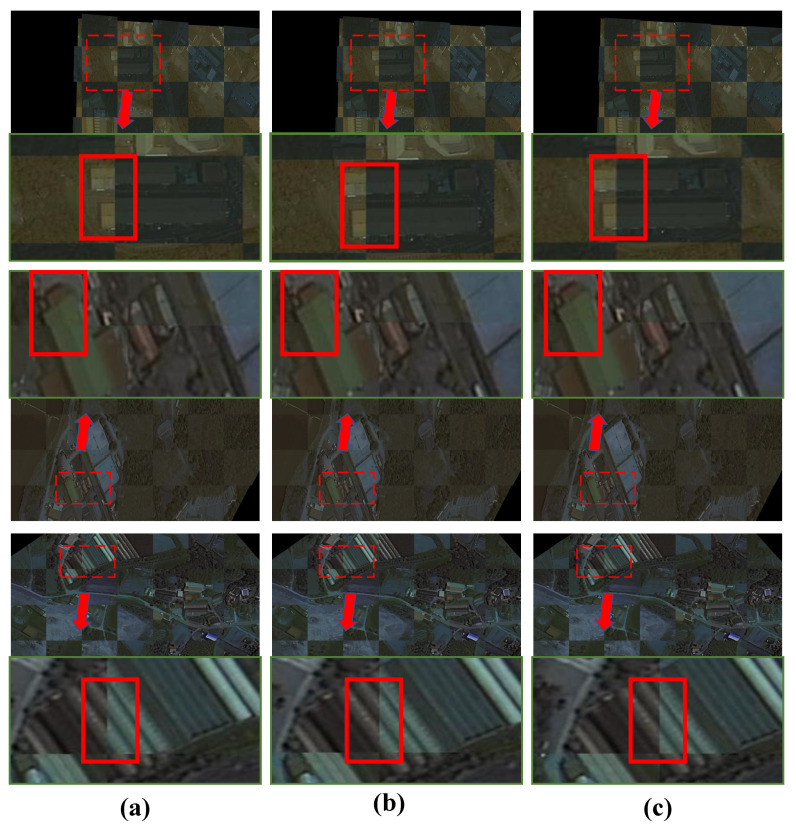
Local comparison of feature-based and region-based models with DVF-NET in S2looking. Tthe solid red box shows the stitching effect of the converted image and the target image: (**a**) DAM-NET; (**b**) Roma; (**c**) DNF-NET.

The registration results are shown in Figure 7 and Figure 8. The traditional SIFT method exhibits global misalignment in the registration of multi-temporal remote sensing images. Overall, all the deep learning-based methods we employed achieved registration, but local misalignment occurred with LoFTR and Roma due to skewed buildings, despite good overall alignment with the target image. The feature-based methods struggled to overcome this challenge. Although DAM-NET performed better than the feature-based methods in handling skewed buildings, it still faced issues with local misalignment, possibly due to training data generation. In contrast, our DVF-NET demonstrated the best registration performance on such images, effectively mitigating the impact of building skewness on the registration results. To comprehensively compare the different methods, we conducted local image comparison experiments on two datasets. As shown in Figure 8 and Figure 9, the proposed model effectively reduced instances of local misalignment in both datasets.

#### 4.1.2. Visual Analytics of Bidirectional Registration Results

**Figure 10:**
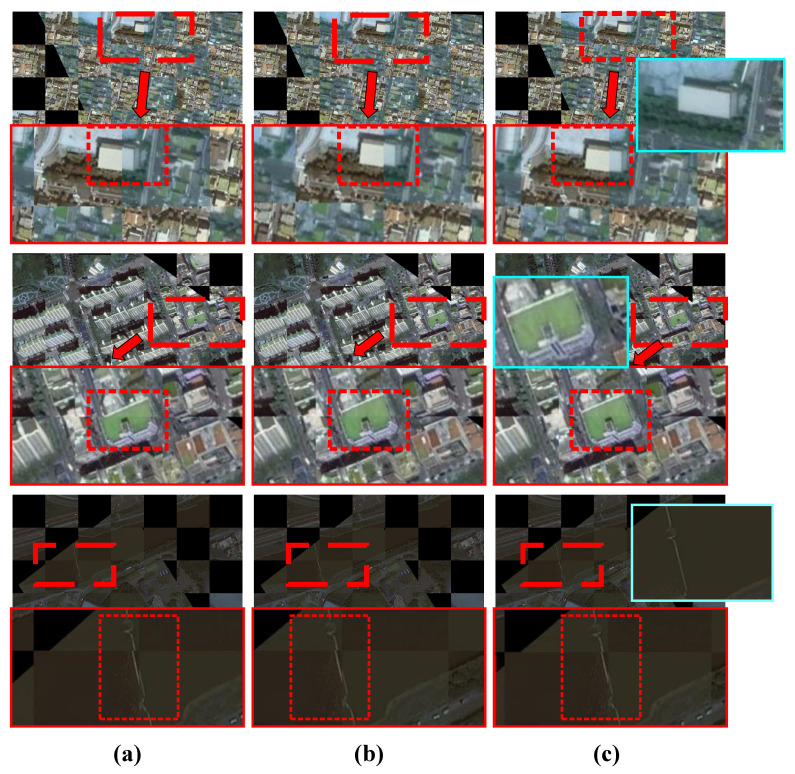
Comparison of reverse registration results in two datasets, where the blue part represents the corresponding section of the source image, and the solid red box shows the stitching effect of the converted image and the target image. The first two are from the Google Earth dataset, and the last one is from the S2looking dataset: (**a**) Roma; (**b**) DAM-NET; (**c**) DVF-NET.

To improve the registration accuracy of the model, it is crucial to ensure high precision in both the forward and reverse directions of registration. According to the reverse registration results shown in Figure 10 and Table 2, DAM-NET and Roma demonstrated excellent performance across both datasets, with differences of less than 5 pixels between them. However, both methods still exhibited instances of local misalignment in both datasets. This highlights the limitations of using a single transformation matrix for registering multi-temporal remote sensing images. Our comparative experiments indicate that our model achieved the best results, significantly improving registration accuracy and reducing local misalignment issues.

#### 4.1.3. Semantic-Based Comparison Results

**Figure 11:**
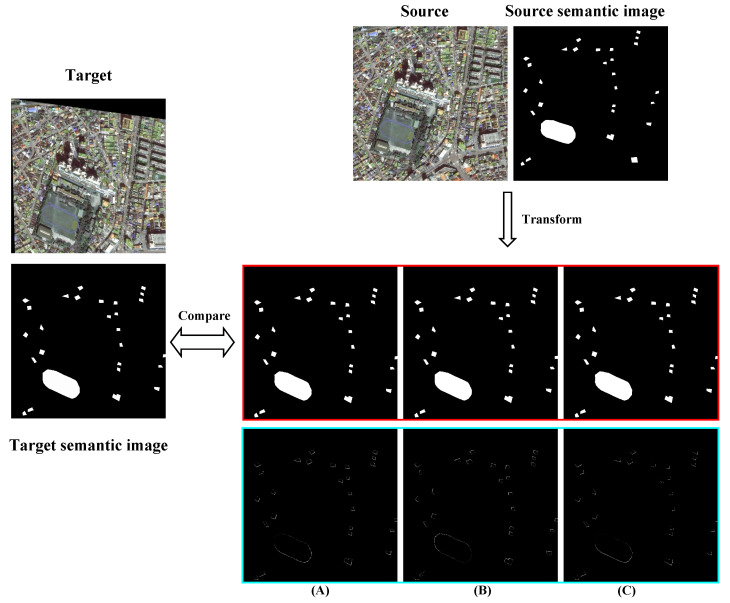
Semantic comparison results of the three methods for low-rise buildings. The red part of the figure represents the semantic map of the converted source image, and the blue part represents the difference between the semantic map of the converted source image and the target image. The smaller the proportion of the white area, the better the registration effect; the results of the percentage of white areas are shown below. (**A**) DAM-NET: 21.49%; (**B**) Roma: 19.26%; (**C**) DVF-NET: 16.30%.

**Figure 12:**
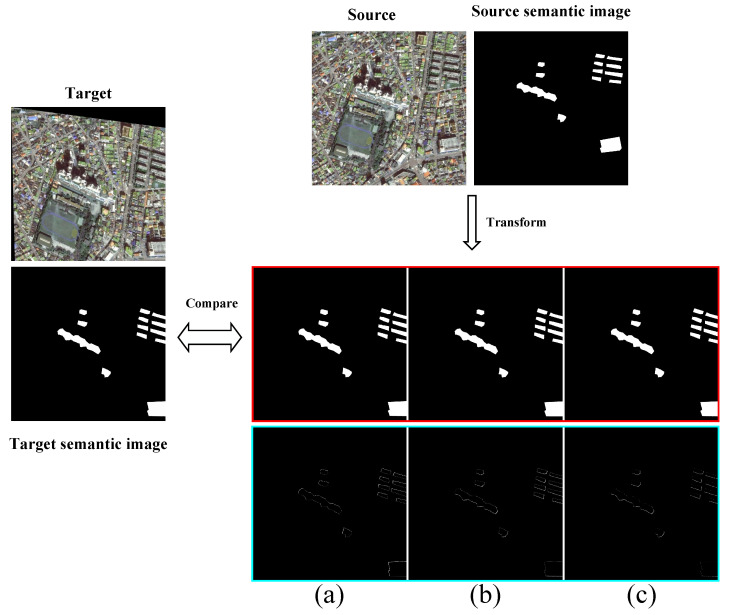
Semantic comparison results of the three methods for tall buildings. The red part of the figure represents the semantic map of the converted source image, and the blue part represents the difference between the semantic map of the converted source image and the target image. The smaller the proportion of the white area, the better the registration effect; the results of the percentage of white areas are shown below. (**a**) DAM-NET: 22.09%; (**b**) Roma: 20.34%; (**c**) DVF-NET: 16.89%.

In this section, we compare the experimental results of high and low buildings in the same image at the pixel level. The comparison results for the low-rise buildings are shown in Figure 11, while those for the high-rise buildings are shown in Figure 12. To compare the registration effects of the different models more clearly, we computed the difference between the registered semantic image and the target semantic image. The obtained results were then differentiated and marked in the figure, showing that our model achieved the best results.

### 4.2. Discussion

#### 4.2.1. The Impact of $\alpha_{1}$, $\alpha_{2}$, and $\alpha_{3}$

Equation (10) is the total loss function of DVF-NET, where $\alpha_{1}$, $\alpha_{2}$, and $\alpha_{3}$ are the weighting coefficients for the three components of the loss. To explore the impact of each component on the registration of dual-phase remote sensing images, we assigned fixed ratio values to $\alpha_{1}$, $\alpha_{2}$, and $\alpha_{3}$ and retrained the network. Validation was performed on the Google Earth dataset, and the experimental results are presented in Table 3. It can be observed that our method achieved the best results when the ratio of $\alpha_{1}$, $\alpha_{2}$, and $\alpha_{3}$ was set to 0.5:0.2:0.3. Additionally, this demonstrates that the influence on the final registration outcome was greatest for $\alpha_{1}$, followed by $\alpha_{3}$ and then $\alpha_{2}$.

#### 4.2.2. The Impact of the Number of Key Points Used for Supervision

**Figure 13:**
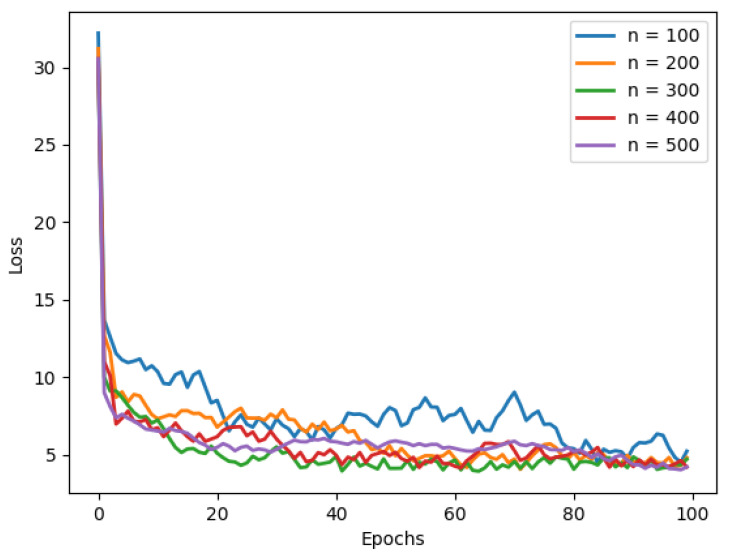
The effect of the number of keypoints on the model’s convergence speed is shown. It can be observed that the green line (*n* = 300) in the diagram converges to the lowest point first.

To investigate the impact of the number of keypoints, *n*, used for network training on network performance, we trained networks using different numbers of keypoints (*n* = 100, 200, 300, 400, 500). In Table 4 and Figure 13, it is observed that the network exhibits optimal convergence speed and accuracy when *n* = 300. Additionally, it is evident that fewer keypoints (*n* < 300) positively affect both convergence speed and accuracy. Conversely, when *n* > 300, there is no significant improvement; instead, a negative impact on convergence speed and accuracy is observed. This phenomenon is primarily attributed to errors inherent in our keypoint extraction process. When the number of keypoints is small (*n* < 300), these errors have a minor impact on the network. However, as the number of keypoints increases (*n* > 300), cumulative errors begin to negatively affect network performance.

#### 4.2.3. Ablation of Different Modules

To explore the effectiveness of each module in DVF-NET, we carried out ablation experiments targeting different components. More specifically, we tested the structural attention module (referred to as STA in the table), the affine prediction module, the projection transformation prediction module, and the combined effects of the latter two modules. For the verification of the structural attention module, we directly utilized the image features extracted by resnet50 while keeping the other processes unchanged. As for verifying how the displacement vector field functions, we directly employed a single displacement vector field to train the model and removed the supervised training of other displacement vector fields. As shown in Table 5, our designed fusion approach notably enhances network performance. Additionally, the structural attention module significantly improves the network’s output results.

## 5. Conclusions

In this paper, we present a network called DVF-NET for dual-temporal remote sensing image registration. The design of this network aims to address the nonlinear distortions caused by significant differences between images by integrating two displacement vector fields, enabling more precise alignment of images. To further enhance the network’s performance, we introduce a Structural Attention Module (SAT), which improves the network’s focus on structural features, thereby increasing the capability and effectiveness of feature extraction. Additionally, we propose a novel loss function design paradigm that combines multiple similarity metrics to provide more comprehensive supervision during the training process. This approach enhances the robustness of the network and significantly improves registration accuracy when handling image pairs with substantial differences. Compared to the benchmark methods evaluated in this study, DVF-NET demonstrates superior performance, validating its potential applications in remote sensing image processing. Overall, DVF-NET provides new ideas and technological advancements for the field of remote sensing image registration.

## Figures and Tables

**Table 1:** Different methods are compared based on experiments on Google Earth dataset and S2looking, where ‘-’ denotes global misalignment. (The best results are indicated in bold).

	Google Earth Datase			S2looking		
Model	PCK < 10	PCK < 8	RMSE	PCK < 10	PCK < 8	RMSE
SIFT	1.2%	0.8%	-	13.4%	11.2%	-
Lofter	71.2%	61.8%	6.4360	97.6%	95.2%	1.5687
Roma	75.0%	63.4%	5.5327	**97.8%**	96.6%	1.3678
DAM	74.8%	63.6%	5.3274	96.5%	95.8%	1.4496
Ours	**77.2%**	**64.2%**	**4.3241**	**97.8%**	**96.8%**	**1.3257**

**Table 2:** Comparison of reverse registration results for two datasets. (The best results are indicated in bold).

	Google Earth Datase			S2looking		
Model	PCK < 10	PCK < 8	RMSE	PCK < 10	PCK < 8	RMSE
Roma	73.8%	61.8%	5.8413	97.2%	96.0%	1.4078
DAM	73.4%	61.4%	5.8174	96.5%	95.2%	1.4796
Ours	**76.6%**	**65.6%**	**4.6212**	**97.6%**	**96.8%**	**1.3757**

**Table 3:** The impact of the three parts of the loss function on the overall performance of the model at fixed ratios of 0.5, 0.3, and 0.2 respectively. (The best results are indicated in bold).

$\alpha_{1}:\alpha_{2}:\alpha_{3}$	PCK < 8	PCK < 10	RMSE
0.5:0.2:0.3	**64.2%**	**77.2%**	**4.3241**
0.3:0.2:0.5	63.8%	76.6%	4.5967
0.2:0.3:0.5	62.0%	75.0%	4.9874
0.2:0.5:0.3	61.6%	74.6%	5.0241
0.3:0.5:0.2	62.8%	76.0%	4.7562
0.5:0.3:0.2	64.0%	76.8%	4.6597

**Table 4:** The influence of the number of keypoints on the model’s result accuracy. (The best results are indicated in bold).

Number of Keypoints	RMSE	PCK < 10
n=100	5.1473	74.8%
n=200	4.9541	75.8%
n=300	**4.4862**	**77.0%**
n=400	4.6926	76.8%
n=500	4.5117	76.4%

**Table 5:** Impact of the different modules of the proposed network. (The best results are indicated in bold).

	STA	Affine	Projection	Field Fusion	PCK < 10	RMSE
1	✔	✔			64.2%	8.2694
2	✔		✔		68.2%	7.8531
3				✔	76.2%	4.9563
4	✔			✔	**77.0%**	**4.5068**

## Data Availability

The original contributions presented in this study are included in the article. Further inquiries can be directed to the corresponding author.
